# Lifestyle and diet in relation to risk of type 2 diabetes in Vietnam: a hospital-based case–control study

**DOI:** 10.1186/s40064-016-2313-3

**Published:** 2016-05-20

**Authors:** Chung T. Nguyen, Ngoc Minh Pham, Dinh V. Tran, Andy H. Lee, Colin W. Binns

**Affiliations:** Department of Epidemiology, National Institute of Hygiene and Epidemiology, Hanoi, Vietnam; Thai Nguyen University of Medicine and Pharmacy, Thai Nguyen, Vietnam; School of Public Health, Curtin University, Perth, WA Australia

**Keywords:** Study protocol, Type 2 diabetes, Tea consumption, Case–control, Risk factors, Lifestyle, Diet, Vietnam

## Abstract

**Background:**

Lifestyle and diet are important determinants of type 2 diabetes (T2D). Their impact on T2D can be evaluated using clinical and epidemiological approaches. Randomised controlled trials are the most rigorous design but expensive to conduct, whereas prospective cohort studies are time-consuming and less powerful for populations with a low incidence of the disease. Case–control studies are considered appropriate in resource-limited settings. A hospital-based case–control study protocol has been developed to investigate the role of lifestyle and dietary factors in T2D aetiology for adults in Vietnam.

**Methods:**

A total of 1100 patients aged 40–65 years (550 T2D cases and 550 controls) will be recruited from a tertiary hospital in Hanoi, the capital city of Vietnam. Cases and controls will be frequency-matched on age (±3 years), gender, and residential location. T2D will be diagnosed according to the 2006 World Health Organisation criteria. Habitual physical activity will be assessed by the Vietnamese version of the International Physical Activity Questionnaire-Short Form. Food and beverage consumption will be ascertained using a Validated Food Frequency Questionnaire, specifically developed for the Vietnamese population. Information on demographic and other personal characteristics will be collected, together with anthropometric and blood pressure measurements. Descriptive statistics and unconditional logistic regression analyses will be performed to examine factors associated with the T2D prevalence.

**Discussion:**

The proposed study will elucidate the role of lifestyle and diet in T2D prevalence among Vietnamese adults. Findings concerning pertinent factors will provide epidemiological evidence for the development of focused interventions, and contribute to the formulation of national policies to prevent and control T2D in Vietnam.

## Background

The prevalence of chronic non-communicable diseases is rising worldwide, with notable increases being observed in low- and middle-income countries (World Health Organisation 2011). As a major non-communicable disease, type 2 diabetes (T2D) poses public health and socioeconomic challenges. In 2013, the estimated number of people with diabetes was 382 million worldwide, of whom 179 million (~47 %) lived in low- and middle-income countries in Asia (Guariguata et al. 2014). Diabetes caused at least USD 548 billion dollars (11 % of the total health spending on adults worldwide) in 2013 (International Diabetes Federation 2013). The aging population and rapid nutritional transition in many Asian countries appear to be the drivers of the growing prevalence of T2D in this region (Council on Foreign Relations 2014). Therefore, it is important to understand the role of lifestyle and diet in the aetiology of T2D in Asia’s emerging economies.

Vietnam is a lower-middle income country in Southeast Asia with a rapidly growing economy. An early study reported that the prevalence of T2D amongst Vietnamese adults was only 1.2 % in 1994 (Quoc et al. 1994). However, a recent systematic review showed that the prevalence of T2D has doubled from 2.7 % in 2002 to 5.4 % in 2012 (Nguyen et al. 2015). This review also found a lack of epidemiological studies addressing the relationship between lifestyle, diet and T2D in Vietnamese adults. For example, a case–control study (Le et al. 2005) suggested that the elevated risk of T2D was associated with the intake of animal protein and meat, but inversely associated with the consumption of carbohydrate and rice, yet the sample size of the study was rather limited (n = 144), and there was lack of consideration of potential confounders and nutrient inter-correlation in this study.

Modifiable lifestyle and dietary factors provide an opportunity for the management, control and prevention of T2D by implementing effective interventions. Despite accumulating epidemiologic evidence of the association between tea and coffee consumption and the risk of T2D (Ding et al. 2014; Yang et al. 2014), few studies have addressed the role of green tea, a popular beverage in Asia, in T2D aetiology (Iso et al. 2006; Odegaard et al. 2008). Similarly, no study has been conducted concerning the effects of coffee consumption in Vietnam, one of the world’s biggest producers of coffee beans. Tea and coffee are the major sources of catechins (Higdon and Frei 2003) and chlorogenic acids (Higdon and Frei 2006), respectively, the so-called phenolic compounds known for their antidiabetic properties (Kim et al. 2016). Because these compounds vary in amount according to tea processing (Shitandi et al. 2012) and coffee variety (Arabica vs. Robusta) (Pham and Preedy 2015), specific types of tea and coffee can produce different effects on the development of T2D.

Given the widespread consumption of green tea and coffee in Vietnam, it is timely to investigate their relation with the T2D risk, along with the effects of lifestyle and other dietary factors. Case–control, cohort studies, and randomised controlled trials (RCTs) provide an ascending hierarchy of levels of evidence (Colditz 2010). RCTs are the most rigorous design but expensive to conduct, whereas prospective cohort studies require a long-term follow up and less powerful for the population with a low incidence of the disease. Case–control studies, being retrospective in nature with a relatively smaller number of participants, are suitable in resource-limited settings (Wang and Attia 2010). In particular, hospital-based case–control studies, with supporting clinical and laboratory services, are less costly and preferable than their community-based counterparts (Schlesselman and Schneiderman 1982). Therefore, a hospital-based case–control study protocol has been developed to ascertain the role of lifestyle and diet in relation to the T2D risk for adults in Vietnam.

Specific objectives of our case–control study are:

1. To determine the association between green tea consumption and risk of T2D.
2. To determine the association between coffee consumption and risk of T2D.
3. To ascertain common foods in the Vietnamese diet (e.g. soy foods, fruits, vegetables, red/processed meat, and white rice-based products) in relation to the T2D risk.
4. To investigate certain macronutrients (e.g. protein, carbohydrate, and fat) and micronutrients (e.g. magnesium, iron, vitamin D, and minerals) in relation to the T2D risk.
5. To determine the association between lifestyle factors (such as smoking, alcohol drinking, and physical activity) and risk of T2D.

## Methods

The study protocol was designed and presented in accordance with the Strengthening the Reporting of Observational Studies in Epidemiology guidelines (Von Elm et al. 2007).

### Study design

This hospital-based case–control study will recruit T2D cases frequency matched to controls by age and sex on a 1:1 ratio in a hospital in northern Vietnam. Data will be obtained by interviewing patients with and without T2D. Medical records and log-books of eligible cases and controls will be searched for the most recent laboratory test results and medical history information.

### Study setting

The proposed study will be undertaken in a tertiary public hospital located in the catchment covering both rural and urban areas of Hanoi City, the capital of Vietnam. This is a general hospital comprising 600 beds, 39 departments/units, and 853 employees. In 2009, there were 317,343 individuals seeking medical care in this hospital. Currently, about 5000 diabetic patients are being monitored and managed in the outpatient department. On average, 30–40 cases of T2D are newly diagnosed every month, allowing the completion of recruitment within 18 months for the target sample size.

### Sample size calculation

A primary objective is to determine the association between the consumption of green tea, a popular beverage in Vietnam, and the risk of T2D. The green tea exposure is thus used to estimate the required sample size. The available data indicated that 70 % of Vietnamese adults consume 1–6 cups of green tea per week (1 cup ~200 mL). We further postulate that the odds ratio (OR) for T2D is 0.66 among individuals drinking 1–6 cups of green tea per week relative to non-drinkers (Iso et al. 2006). With frequency matching by age and sex between cases and controls on a 1:1 ratio, a total of 1080 subjects (540 T2D cases and 540 controls) will be required to attain 90 % statistical power for such effect size at 5 % level of significance (Schlesselman 1974). To compensate for potential refusal and withdrawal, 1100 subjects (550 cases and 550 controls) will be recruited. The proposed sample size should be sufficient to detect moderate effect sizes for other dietary and lifestyle factors such as coffee consumption and physical activity levels.

### Selection of cases

Potential participants will be identified by searching the medical records of the Department of Endocrinology. The hospital uses the 2006 World Health Organisation (WHO) diagnostic criteria for T2D (WHO 2006) according to the levels of fasting plasma glucose and/or 2 h-oral glucose tolerance test (2 h-OGTT). Consecutive patients newly diagnosed with T2D will be assessed for eligibility when they come to the hospital for treatment. An appointment will be arranged for a personal interview if the patient meet the following selection criteria, agree to participate and can voluntarily provide informed consent.

#### Inclusion criteria

- Aged 40–65 years;
- Diagnosed with T2D within 4 weeks before the interview; and
- Being able to undertake a 45-min face-to-face interview including measurements.

#### Exclusion criteria

- Deemed too ill to participate by the medical doctors;
- Diagnosed with T2D more than 4 weeks before the interview;
- In long-term modification of diet due to any reason; and
- Non-residents, tourists or foreigners.

### Selection of controls

Controls will be individuals seeking medical care at the outpatient clinics of the departments of orthopaedics, dental, ear, nose and throat, and gastroenterology, who are generally healthy and unlikely to share similar risk profiles as the cases. As a partner of the national diabetes prevention program, all patients attending the hospital outpatient clinics will be screened and tested for T2D by administering fasting glucose test and/or 2 h-OGTT, results of which are entered into the patient log-book and medical record on the day of their visit. The physician will then confirm their diabetes-free status on the basis of the 2006 WHO diagnostic criteria. Eligible controls, being frequency-matched to cases on sex, age (±3 years), and residential location, will be recruited at the same data collection period as the cases. They will be interviewed by the research assistant using the same questionnaire upon their consent to participate in the study.

#### Inclusion criteria

- Individuals who attend the hospital outpatient clinics due to minor health problems;
- Whose plasma glucose levels are within the normal range based on the 2006 WHO diagnostic criteria;
- At the same age group as cases (±3 years); and
- Reside in the same catchment area (metropolitan Hanoi) as the cases.

#### Exclusion criteria

- Non-diabetic patients with malignant cancers;
- Outside the desired age range;
- Deemed too ill to participate by the medical doctors;
- In long-term modification of diet due to any reason; and
- Non-residents, tourists or foreigners.

### Recruitment procedure

On a typical day, the research team members will be present at the hospital outpatient clinics when patient registration begins in the early morning. Eligible T2D patients who agree to participate will be referred by their physicians to the interviewer. The distribution of sex and age of enrolled cases will be continuously updated before the selection and recruitment of controls in the afternoon, who are frequency-matched to cases on sex, age (±3 years), and residential location. After being briefed about the study purpose, each participant will be asked to sign an informed consent form, followed by a face-to-face interview and anthropometric measurements that take on average 45 min to complete. This recruitment process, expected to take 18 months, will continue daily until the desired sample size of n = 1100 is reached.

### Data collection

A structured questionnaire will be administered to each consented participant during the face-to-face interview. This questionnaire comprises several sections related to personal, demographic and residential/contact details, dietary intake, habitual physical activity and lifestyle, followed by clinical information and anthropometric measurements.

#### Dietary assessment

Dietary habits over the three preceding years will be ascertained using a validated semi-quantitative Food Frequency Questionnaire (FFQ), specifically developed for the Vietnamese population (Tran et al. 2013a). This FFQ records the intake of commonly consumed food and beverage items in Vietnam, which are classified into 15 major food groups (soybean products, tomato products, vegetables, fresh fruits, cereal, meat, seafood, egg, cooking oil, seasoning, milk, alcohol, tea, coffee, and juice). The frequency of consumption is documented according to month, week, or day. A portion size (small, standard or large) is estimated via a picture book taken from a previous study (Kusama et al. 2005). Dietary intakes of the 128 food and beverage items, energy and selected nutrients will be computed, with reference to the standard tables of food composition in Vietnam (Khan 2007).

#### Physical activity assessment

Physical activity levels including vigorous and moderate activities as well as walking before the onset of T2D will be assessed by the Vietnamese version of the International Physical Activity Questionnaire-Short Form (Tran et al. 2013b). Sitting time will be inquired about the amount of time spent using computers, watching television, eating meals, reading newspapers/book and sitting on motorbikes, in vehicle or train. Information about sleeping duration will also be solicited.

#### Lifestyle assessment

Questions on smoking and alcohol drinking are taken from the WHO’s STEPwise approach to non-communicable disease risk factor surveillance (WHO 2008) and adapted to suit the Vietnamese context. Past and current smokers will be asked to report their average number of cigarettes smoked per day or per week, as well as number of months or years of smoking. Never smokers will be asked whether they are exposed to passive smoking at work, public places or at home, and if exposed, the frequency of exposure will be recorded.

#### Clinical data extraction

Clinical and biochemical laboratory results including fasting plasma glucose, 2 h-OGTT, HbA1c and lipid profile (triglycerides, cholesterol levels) will be extracted from patient log-books on the date of interview. Comorbidities will be inquired by the interviewer and cross-verified against medical records. Family history of diabetes (type 1 or type 2) will be recorded if applicable.

#### Measurements

Anthropometric measurements including height, weight, waist and hip circumferences, will be measured by the research team following standard protocols. Standing height is measured using a stadiometer bar, without shoes, with shoulders in a relaxed position and arms hanging freely, and recorded to the nearest 0.1 cm. Body weight is measured when wearing light clothing without shoes on a digital electronic weighing scale (Tanita BC-601, range 0.1–150 kg) and recorded to the nearest 100 g. Body mass index is then calculated as the weight (kg) divided by height (m) squared. Waist circumference (WC) is measured as the minimal abdominal circumference between the lower edge of the subcostal ridge and the iliac crests. Hip circumference is determined using tape as the broadest buttock circumference. The waist-hip ratio is then calculated as WC (cm) divided by hip circumference (cm), as is the computation of waist-height ratio.

Blood pressure and pulse rate will also be taken to double check those written in medical records. They are measured twice in a sitting position after the participant has rested for at least 5 min. Percent body fat and central adiposity levels will be measured by Bioelectrical Impedance Analysis (Kyle et al. 2004) using a BC-601 scale (Tanita Corporation, Tokyo, Japan).

### Data management

The transcribed questionnaires will be checked by the Principal Investigator (first author). Missing, inconsistent, or illogical information will be clarified with notes and subsequently rectified. Only the Principal Investigator is allowed to correct erroneous information. An audit trail will be kept for data collection and manual verification. The hard copy questionnaires will be temporarily stored in a hospital office accessible by the Principal Investigator only. Data will be entered into Epi-data (EpiData Association 2005) on a daily basis, so that logical errors, missing information and incorrect coding can be immediately identified. Thereafter, range checking and outliers detection will be performed within Epi-data to ensure the quality of data entered. Outliers will be screened using the Tukey’s method (boxplot), diagnostic graphs and residual measures (Osborne 2010). Once data cleaning is completed, the electronic dataset will be securely stored in a password-protected computer at the National Institute of Hygiene and Epidemiology, Vietnam.

### Statistical analysis

The outcome variable is dichotomous, i.e., presence or absence of T2D. Plausible dietary and lifestyle factors include green tea and coffee consumption, certain food items and nutrients, physical activity, smoking, and alcohol drinking. Additional potential variables of interest include demographics (e.g. marital status, occupation, and education level), clinical information (systolic and diastolic blood pressure as well as pulse rate) and anthropometrics (e.g. height, weight, waist and hip circumferences, percent body fat, and central adiposity).

The dataset will be exported to the SPSS package (version 20.0; SPSS Inc., Chicago, IL, USA) for statistical analyses. Descriptive statistics will be initially performed, followed by multivariable regression analysis. Specifically, characteristics of exposure and confounding variables will be compared between case and control groups using two-sample t test or Wilcoxon rank-sum test for continuous variables, and Chi square test for categorical variables. Dietary patterns will be derived using principal component analysis (Dunteman 1989) based on energy-adjusted intakes of food and beverage items with reference to a density method (amount of food intake per 1000 kcal of energy) (Willett 2012). Scores of each dietary pattern will be categorised into quartiles. Associations between exposure variables and risk of T2D will be assessed using unconditional logistic regression models. Adjusted OR and 95 % confidence intervals for T2D risk with respect to each factor of interest will be presented, taking into account plausible confounding variables which are identified based on prior knowledge, causal diagram, and change-in-estimate strategies (Greenland and Pearce 2015; Greenland 1989). Interaction of potential effect modifiers will be ascertained using the likelihood ratio test. A test for linear trend for each exposure variable will also be performed to assess its dose–response relationship with the T2D risk. A P value <0.05 is considered statistically significant.

### Ethical considerations

All participants will provide written informed consent prior to the conduct of interview and measurements. Ethical approval of this protocol has been obtained from the Human Research Ethics Committee of Curtin University (Approval Number of HR105/2013). Agreement for patient recruitment and data collection has been given by the hospital authority involved in this study.

## Discussion

A study protocol has been developed for a hospital-based case–control study of T2D involving 1100 participants aged 40–65 years in Vietnam. This study will address whether the consumptions of green tea and coffee are associated with the risk of T2D. It will also assess lifestyle and other dietary factors which may be related to the development of T2D in the Vietnamese population. Previous findings on risk and protective factors of T2D were predominantly reported from high-income countries (Jeon et al. 2007; van Dam and Hu 2005; Huxley et al. 2009; Willi et al. 2007; Alhazmi et al. 2014). Notwithstanding the importance of these findings, further epidemiological evidence from developing nations is needed to ascertain the apparent relationships, yet few investigations have been undertaken using the case–control design which is suitable in resource-limited settings (Wang et al. 2002; Radzeviciene and Ostrauskas 2009; Naja et al. 2012; Radzevičienė and Ostrauskas 2012, 2013). To the best of our knowledge, the present proposal is the first case–control study of lifestyle and diet in relation to the risk of T2D among Vietnamese adults using validated instruments and administered via face-to-face interviews. The findings are expected to contribute to the development of evidence-based guidelines for individuals at risk of T2D, with the ultimate goal to control and prevent this emerging chronic disease in Vietnam. Moreover, the study is envisaged to facilitate future replications in other low- and middle-income countries in Asia.

There are several strengths of the proposed study, namely, a relatively large sample size to assess different exposures, use of validated instruments for diet and lifestyle assessment, ascertainment of exposure variables via structured interviews, and uniform measurements of clinical and biochemical parameters across case and control groups. Special efforts will be made to increase response rates and minimise withdrawal of participants. However, some limitations warrant attention. Case–control designs are inherently retrospective in nature, as such they are susceptible to biases. For instance, selection bias may arise if only severely ill patients are recruited and the controls are drawn from a different reference population. For this reason, we will select newly diagnosed (incident) T2D patients, while non-diabetic controls are recruited from the outpatient clinics of the same hospital, who are matched to the cases in terms of residential location but are unlikely to share the same risk profiles. Moreover, the hospital serves the entire catchment covering both rural and urban areas of Hanoi, so that our sample of participants should be representative of the underlying adult population. Another potential drawback is recall bias (Armenian 2009), which may lead to spurious associations between exposures and the outcome of interest (Gordis 2000). In this study, a consistent recruitment and interview process is adopted throughout the data collection period and applied to both case and control groups, particularly the use of interviewer-administered questionnaires, which should reduce recall error and bias between the two groups.

## Abbreviations

T2D: type 2 diabetes
WHO: World Health Organisation
FFQ: Food Frequency Questionnaire
RCT: randomised controlled trials
MET: metabolic equivalent
2 h-OGTT: 2 h-oral glucose tolerance test
OR: odds ratio
WC: waist circumference
